# An Experimental Study and FEM-Based Analysis for Road Safety Barriers: Additively Manufactured PLA–Geopolymer Hybrid Composites

**DOI:** 10.3390/polym18080905

**Published:** 2026-04-08

**Authors:** Muhammed Fatih Yentimur, Oğuzhan Akarsu, Cem Alparslan, Tuba Kütük-Sert, Şenol Bayraktar, Abdulkadir Cüneyt Aydin, Ahmet Tortum

**Affiliations:** 1 Department of Civil Engineering, Faculty of Engineering and Architecture, Recep Tayyip Erdogan University, 53100 Rize, Türkiye; muhammedfatih.yentimur@erdogan.edu.tr (M.F.Y.); tuba.kutuk@erdogan.edu.tr (T.K.-S.); 2 Department of Civil Engineering, Faculty of Engineering, Atatürk University, 25000 Erzurum, Türkiye; oguzhnakarsu@gmail.com (O.A.); acaydin@atauni.edu.tr (A.C.A.); atortum@atauni.edu.tr (A.T.); 3 Department of Mechanical Engineering, Faculty of Engineering and Architecture, Recep Tayyip Erdogan University, 53100 Rize, Türkiye; senol.bayraktar@erdogan.edu.tr

**Keywords:** additive manufacturing, transportation, hybrid polymer–inorganic composites, energy absorption, finite element analysis, road safety barrier, mass efficiency

## Abstract

This study investigates the impact response and energy absorption performance of additively manufactured PLA–geopolymer hybrid composites for potential application in road safety barriers. Hybrid Charpy specimens were fabricated with three different infill densities (20%, 60%, and 100%), combining a 3D-printed PLA outer shell with a geopolymer core. Charpy impact tests were conducted in accordance with ISO 179-1 and ASTM D6110, and the absorbed energy, specific energy absorption, and mass efficiency were determined experimentally. A phase-based analytical model was also used to estimate elastic energy contributions, while fracture surfaces were examined to identify infill-dependent damage mechanisms. To extend the material-level findings to an engineering-scale application, the observed trends were transferred to a New Jersey-type road safety barrier model and evaluated using ANSYS Explicit Dynamics. The results showed that infill density strongly affects fracture behavior and energy dissipation performance, with 60% infill providing the most balanced response in terms of energy absorption and mass/material efficiency. The originality of the present study lies in going beyond a material-scale investigation of the impact behavior of additively manufactured PLA–geopolymer hybrid structures by integrally correlating the experimental Charpy results with a theoretical energy-based framework, fracture-surface observations, and explicit dynamic finite element analysis of a New Jersey-type road safety barrier model.

## 1. Introduction

Additive manufacturing (AM) is an innovative manufacturing technology that enables the production of components with complex geometries with high precision and minimal material waste [1,2,3]. Compared to traditional manufacturing methods such as machining or molding, additive manufacturing offers significant advantages in terms of energy efficiency, design freedom, and material optimization [4,5]. Beyond geometric freedom, FDM-based additive manufacturing also enables microstructural tailoring through controlled deposition paths, layer-by-layer assembly, and shear-flow-induced alignment phenomena, thereby expanding its potential for the development of high-performance functional materials. Recent work has shown that such process-induced structural organization can significantly enhance anisotropic functional performance, highlighting that FDM can serve not only as a shaping technique but also as a platform for structure–property engineering in advanced material systems [6]. In addition, recent studies on additively manufactured engineering components have shown that their reliable structural application requires combined experimental validation and dynamic numerical analysis, particularly when complex printed geometries are subjected to service-relevant loading conditions [7]. Such integrated experimental–computational approaches provide an important framework for assessing structural response and design reliability in AM-enabled functional systems. Particularly in line with sustainable production goals, integrating reusable, recyclable, or biodegradable materials into these technologies is becoming increasingly important [8]. In recent years, biopolymer and inorganic matrix-based composites, developed to reduce environmental impact, stand out not only for their low carbon footprint but also for their engineering performance characteristics such as strength, rigidity, and energy absorption capabilities [9,10,11]. In this context, combining polymers such as bio-based polylactic acid (PLA) and geopolymer-based inorganic binders in hybrid systems enables the development of environmentally friendly, high-strength, and thermally stable composites [12,13,14]. The development of such hybrid structures demonstrates an orientation aligned with the Sustainable Development Goals (SDGs) in terms of both material circularity and carbon emission reduction. Thus, additive manufacturing has become a platform directly related not only to production efficiency but also to the principles of the circular economy and green engineering.

Traffic accidents cause the death of more than 1 million people every year and continue to be a significant socio-economic burden [15,16]. These traffic accidents can be single-vehicle, vehicle-to-vehicle, or vehicle-to-pedestrian collisions, or they can occur as a result of vehicles leaving the road and exiting their lane uncontrollably. A significant portion of serious consequences is associated with “leaving the road” and “lane violation” scenarios, such as a vehicle leaving the carriageway and colliding with roadside objects or crossing the median into the opposite lane [17,18,19]. In this context, road safety barriers (RSBs) are considered the last line of defense, limiting and/or redirecting the movement of a vehicle moving in the wrong direction or losing control, and aiming to reduce the severity of a collision through vehicle stability, passenger compartment accelerations, and the system’s capacity to manage kinetic energy [20,21]. Longitudinal RSBs, in particular, aim to mitigate the consequences of collisions by providing a controlled redirection path to prevent vehicles from crossing into the opposite lane or hazardous areas; however, this effectiveness directly depends on the mechanisms by which collision energy is dissipated and how forces are transferred across the barrier system [22,23,24]. Within the longitudinal RSB family, rigid concrete barriers with a New Jersey profile are widely used in median strips, bridge approaches and curbs, inner/outer edges of curves, and tunnel entrances and exits due to their strength, ease of maintenance, and ability to redirect traffic with limited lateral displacement [25]. This barrier geometry aims to reduce the risk of rollover by regulating the wheel-barrier interaction and body movement of the vehicle at shallow collision angles; however, due to the nature of rigid barriers, it is possible that collision forces are transmitted at higher peak values and the “intrinsic” energy dissipation capacity of the barrier system remains limited [26]. Due to the rigid mass behavior, the energy absorption achieved by the system through its own deformation is limited and can increase the deformation on the vehicle/person during an impact [27,28]. These limitations have led to an increased trend towards composite RSBs in recent years—for example, polymer, fiber-reinforced polymer (FRP), or polymer-coated hybrid solutions; the aim is to provide additional damping and controlled crushing/plastic deformation at the contact area while maintaining steering capability [24,29]. More broadly, studies on lightweight transportation composites have demonstrated that impact-energy management and structural stiffness depend strongly on the optimization of structural architecture, including laminate configuration and core–skin interaction. Although developed for different application domains, these design principles provide a useful conceptual background for interpreting shell–core hybrid systems designed for controlled deformation and energy dissipation [30,31]. Recent studies on the design, structural optimization, and high-fidelity finite element modeling of multilayer composite panels in hybrid road safety structures highlight the decisive role of internal architecture and layered structure representation on stiffness distribution, energy management, and dynamic response. In particular, the combined use of design-optimization approaches with high-fidelity FEM and dynamic analysis frameworks in multilayer composite panel-based hybrid vehicle structures provides a significant methodological background for interpreting the behavior of shell–core-based hybrid systems, even if they belong to different application areas. This context also provides conceptual support for the internal architectural representation and numerical evaluation at the engineering scale of the PLA–geopolymer hybrid structure addressed in the present study [32,33].

PLA is a biodegradable thermoplastic derived from renewable resources such as corn starch or sugarcane. Widely used in additive manufacturing technologies, particularly fused deposition modeling (FDM), PLA has gained considerable attention in sustainable production due to its low environmental impact and ease of processing [34,35]. However, its impact resistance, thermal stability, and fracture toughness are generally insufficient for demanding engineering applications when used alone [36]. Therefore, increasing research has focused on the development of hybrid PLA-based composites incorporating fibers, nanoparticles, or inorganic phases to enhance overall material performance [37,38,39]. In hybrid structural concepts, PLA can be considered a promising FDM-compatible polymer phase because it enables precise control of internal architecture, lightweight fabrication, and tunable energy dissipation behavior. Within such shell–core configurations, the PLA phase may serve as an outer energy-dissipating shell, while the inorganic core contributes stiffness and dimensional support. From this perspective, the relevance of PLA lies primarily in its manufacturability, controllable deformation behavior, and suitability for hybrid structural design, rather than in the assumption that neat PLA alone would be sufficient for long-term roadside service. Nevertheless, the long-term environmental stability of PLA under complex service conditions, including UV exposure, moisture, and thermal cycling, remains an important issue and should be addressed in future studies through durability-focused investigations and, where necessary, the use of stabilized or reinforced PLA-based formulations.

Geopolymers are inorganic polymers formed by alkali activation of aluminosilicate-based sources (such as fly ash, metakaolin, or slag), possessing high temperature resistance and hardness coefficients [40,41]. With CO_2_ emissions up to 80% lower than traditional Portland cement, geopolymers stand out as environmentally friendly binder systems [42,43,44]. However, geopolymers have disadvantages such as exhibiting brittle behavior and offering limited energy absorption under dynamic loads [45,46,47,48]. At this point, PLA–geopolymer hybrid composites have the potential to create a new class of materials by combining the complementary properties of the two materials. When the flexibility and formability of PLA are combined with the high stiffness and thermal stability of the geopolymer, a composite structure with high impact resistance, enhanced thermal stability, and recyclability can be obtained [12,49]. Despite this, studies on the impact behavior and energy absorption performance of PLA–geopolymer hybrids produced by additive manufacturing methods are quite limited in the literature. In this context, geopolymer-based inorganic phases are attracting attention due to their environmentally friendly structures, low carbon footprints, and high temperature resistance. Ricciotti et al. (2023) investigated the consistency properties of geopolymers for extrusion-based 3D printing and the relationships between printing parameters and microstructure and stated that these materials can be successfully integrated into 3D printing processes [50]. Similarly, Shilar et al. (2023), summarizing the current status of 3D-printed geopolymer composites, highlighted that they offer high stiffness, fire resistance, and environmental advantages, but still suffer from shortcomings such as brittle behavior and limited energy dissipation capacity under impact [51]. On the PLA side, Caminero et al. (2019) showed that graphene nanoplatelet-reinforced PLA composites improved mechanical performance and impact resistance [52]. Desole et al. (2024) evaluated the energy absorption capacity of PLA-based metamaterials by impact testing and revealed the effect of different cellular structure designs on energy absorption behavior [53]. In addition, Kazantseva et al. (2025) investigated geometric strategies that optimize the impact energy of 3D-printed PLA specimens in biomimetic structure forms [54]. On the concrete barrier side, Kömürlü (2021) investigated the effect of applying a polyurethane-based thermoset foam “energy-absorbing primer” to the impact surface of rigid concrete barriers used against rockfalls on the damping of impact energy within the coating and the resulting shear and body damage behavior using Charpy-based laboratory experiments [55]. Aung and Jarasjarungkiat (2024) evaluated the effects of natural rubber sheet coating and waste tire-derived rubber additive on impact energy damping and damage reduction on concrete barriers using numerical and experimental approaches [56]. Fallon and McShane (2019) evaluated the potential of a spray elastomer (polyurea/polyurethane hybrid) coating applied to a concrete surface to reduce the energy transferred to the concrete during impact by dissipating a significant portion of the energy through deformation and viscoelastic damping within the coating, using experiments and a finite element approach [57].

Current studies indicate that research largely focuses on improving mechanical properties by adding powdered geopolymer additives to PLA matrices or evaluating the adhesion behavior of geopolymer surface coatings. Nevertheless, the effects of AM parameters and layer orientation, as well as geopolymer ratio and PLA–geopolymer interface interaction, on impact behavior and energy absorption capacity have not yet been systematically elucidated—a critical gap given that collision energy management and damage tolerance are primary design requirements for road safety barriers (RSBs). Current literature has predominantly focused on improving the mechanical properties of PLA matrices through the incorporation of geopolymer-based additives or on evaluating the adhesion behavior of geopolymer coatings. In contrast, the energy absorption behavior and fracture mechanisms of additively manufactured PLA–geopolymer shell–core hybrids under impact, as well as the way this behavior can be transferred to an engineering-scale application such as a road safety barrier, have not yet been clearly established. The originality of the present study can be summarized in three main aspects. First, the impact behavior and mass efficiency of PLA–geopolymer hybrid specimens with different infill densities were experimentally compared under a constant infill pattern. Second, the experimental findings were interpreted together with a phase-based theoretical energy approach and fracture-surface observations, allowing the infill-density-dependent damage mechanisms to be clarified. Third, the trends identified at the material scale were transferred to a New Jersey-type road safety barrier model and evaluated at the engineering scale through explicit dynamic finite element analysis. In this way, the study presents, through a multi-level approach, the potential of PLA–geopolymer hybrids for use in energy-dissipation-oriented barrier components.

## 2. Materials and Methods

### 2.1. Materials: PLA Filament and Geopolymer Composition

In this study, two main components were used in the preparation of hybrid composites: PLA filament and geopolymer concrete. PLA, a commercially available filament for FDM (Fused Deposition Modeling)-type 3D printers, was supplied as ESUN [58] brand filament wound on a spool with a diameter of 1.75 mm (Table 1).

Geopolymer concrete phase was prepared based on a mixture of fly ash and blast furnace slag. The geopolymer mixture used in this study is given in Table 2.

**Figure 1:**
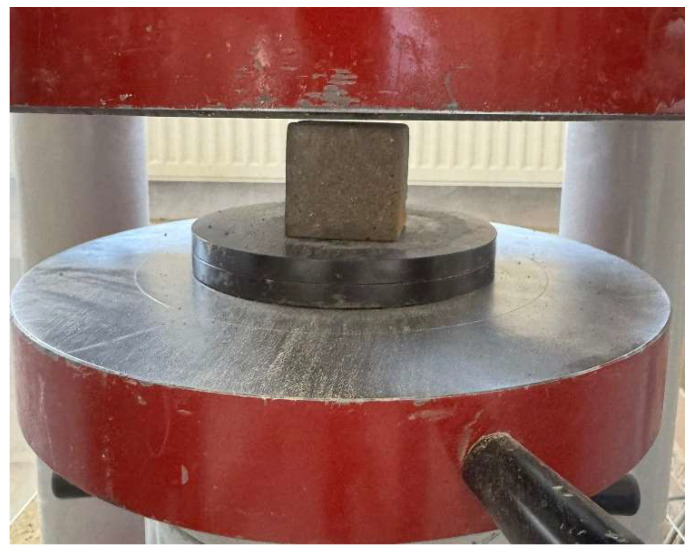
Compression strength test.

The activation solution was prepared from a mixture of sodiumsilicate and sodium hydroxide; the addition of a plasticizer was used to improve the workability and ensure the homogeneity of the mixture [60]. All components were weighed in the specified proportions and mixed with a low-speed mixer for 5 min until the mixture was homogenized. The resulting mixture was poured into standard Charpy test specimen molds (55 mm × 10 mm × 10 mm) and cured at room temperature for 28 days to allow complete polymerization and strength development [61]. These conditions were chosen to ensure that the geopolymer concrete acquired sufficient mechanical strength and microstructural stability. To determine the mechanical properties of geopolymer concrete, compressive strength tests were performed on standard cube specimens with dimensions of 50 mm × 50 mm × 50 mm [62]. Tests were conducted in accordance with the EN 12390-4 standard and were performed after the completion of a 28-day curing period [63]. The tests were performed on a universal compression testing machine with a capacity of 2000 kN (Figure 1). The maximum load values at fracture were recorded, and the compressive strength was calculated for each sample.

### 2.2. Additive Manufacturing and Fabrication of PLA Infill Structures

PLA-based samples were produced using a Bambu Lab X1 Carbon desktop 3D printer (Bambu Lab, Shenzhen, China) (Table 3) [64]. The geometric models were prepared in SolidWorks 2018, converted into STL format, and processed in Bambu Studio to generate the G-code for printing. The main printing parameters used in specimen fabrication are summarized in Table 4.

Table 3 summarizes the technical specifications of the Bambu Lab X1 Carbon printer used in this study. The specimen geometries were modeled in SolidWorks 2018, converted to STL format, and sliced in Bambu Studio prior to printing.

**Figure 2:**
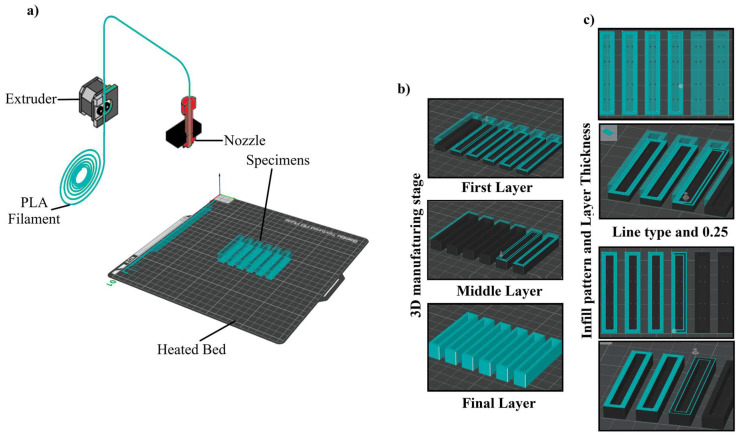
3D printing process performed with the Bambu Lab X1 Carbon system: (**a**) routing of PLA filament from the extruder to the hot end, (**b**) layer-based production stages, and (**c**) schematic representation of the infill pattern and layer thickness.

The specimens required for the Charpy impact test were produced using a Bambu Lab X1 Carbon 3D printer. The outer surfaces of the specimens were printed entirely with PLA filament, while the inner parts were left empty and subsequently filled with geopolymer mortar. Thus, a hybrid structure consisting of an organic polymer shell and an inorganic core was obtained. During the production process, infill densities were determined at three different levels (20%, 60%, and 100%). In this study, the internal infill pattern was kept constant among the additive manufacturing variables, and only line-type infill architecture was used. The only main parameter changed within the experimental design was the infill ratio (20%, 60%, and 100%). Therefore, the comparison of alternative infill patterns was excluded from the scope of this study. Figure 2c presents a schematic representation of the line-type infill architecture used in this study. These parameters were chosen to evaluate the effect of layer density and internal volume geometry on energy dissipation under impact. The geometry of the Charpy specimens was designed according to standard dimensions, and the 3D printing parameters (layer thickness, printing speed, nozzle temperature, and infill pattern) were optimized through preliminary tests (Table 4).

**Figure 3:**
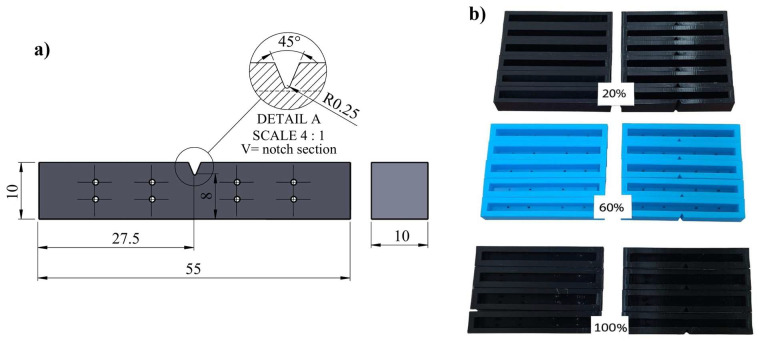
Design of Charpy impact specimens and PLA outer shell specimens produced at different fill ratios, (**a**) technical drawing, and (**b**) PLA specimens with 20%, 60%, and 100% fill ratios.

The PLA samples produced were then filled with a pre-prepared geopolymer mixture to complete the composite structure. Thus, the synergistic behavior of the material in terms of impact resistance was aimed for by providing polymeric energy dissipation in the outer shell and high stiffness contribution of the geopolymer in the inner region. The technical drawing of the samples produced with different filling ratios and the Charpy test specimen is shown in Figure 3. Charpy impact test specimens were prepared in accordance with ISO 179-1:2023(E) and ASTM D6110-18 standards (Figure 3a) [65,66,67]. The samples are 55 × 10 × 10 mm in size, with a V-notch in the center at a 45° angle and a peak radius of 0.25 mm. Each sample type was produced in six replicates with different infill percentages (20%, 60%, and 100%). 3D-printed PLA was used as the outer shell material, and the inner voids were filled with geopolymer mortar to obtain a hybrid composite structure.

### 2.3. Charpy Impact Test Setup (Notched and Unnotched)

Charpy impact tests were performed using an Instron CEAST 9050 Impact Pendulum (Instron, Italy) in accordance with ISO 179-1:2010(E) and ASTM D6110-18 standards [68]. This device is a pendulum impact testing system that allows for the safe and repeatable determination of the impact strength of plastic and composite materials. The CEAST 9050 model has an energy capacity in the range of 0.5–50 J and supports all Charpy, Izod, and Tensile Impact testing methods. The test system is designed to measure the energy absorption capacity and toughness behavior of the material during impact. Samples were tested in the Charpy configuration of the device; the portion of the pendulum energy absorbed by the sample during the fracture process was automatically recorded. The samples used in these tests have the dimensions given in Figure 3, with one group prepared with a V-notch (45° angle, R = 0.25 mm apex radius, 2 mm depth) and the other group without a notch. Six repetitions (n = 6) were performed for each infill percentage (20%, 60%, and 100%). During impact tests, the energy absorbed by the sample (U) was automatically recorded by the device. Using these values, the specific energy absorption (SEA = U/m) and impact resistance (R = U/A) parameters were calculated. These parameters were used to comparatively evaluate the energy absorption performance of hybrid structures with different infill ratios under impact.

**Figure 4:**
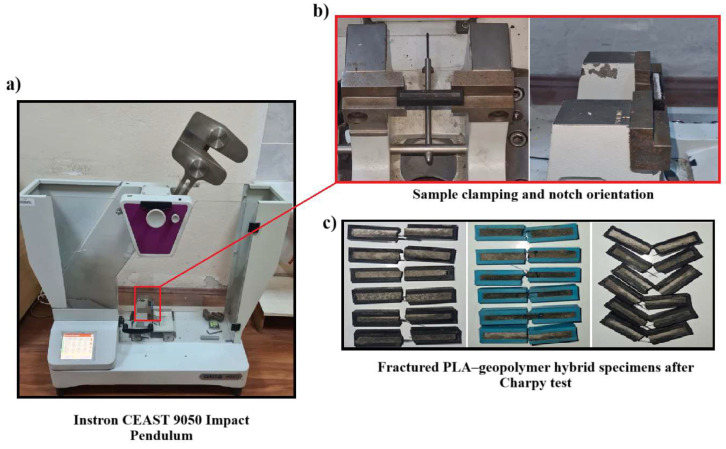
Instron CEAST 9050 impact tester and test setup, (**a**) general view of the device, (**b**) sample placement in the test grips and notch orientation, and (**c**) PLA–geopolymer hybrid samples produced at different filling ratios and imaged after fracture.

The samples were placed horizontally in the sample holder jaws of the device before testing; in notched samples, the notch surface was positioned facing the direction of the pendulum’s impact. The gap between the sample support and the pendulum was set to 40 mm, in accordance with the standard. The pendulum was released aligned with the center of the sample, and the deformation behavior during impact was recorded via high-speed sensors. Figure 4 shows the method of attaching the sample to the device and the test setup during the impact test.

### 2.4. Theoretical Modeling of Impact Energy

The phase-based analytical model used in this study was not intended as a fracture model capable of quantitatively and fully representing the energy behavior of hybrid specimens composed of a PLA outer shell and a geopolymer core under Charpy impact loading. Rather, it was employed as a first-order analytical estimation approach based on elastic contributions. The model considers the mechanical properties of both phases in the hybrid cross-section, their elastic energy storage capacities, and the effect of infill density on the effective PLA volume. From this perspective, the main purpose of the model is not to predict the absolute impact energy with high accuracy, but to provide a physically based interpretation of the overall trend associated with changes in infill density.

Within the scope of the model, it was assumed that PLA and geopolymer phases exhibit isotropic, homogeneous, and linear elastic behavior in the pre-fracture loading regime, and that energy storage during impact occurs primarily through elastic deformation. In addition, the infill ratio was considered to affect only the effective volume of the PLA phase, while the volume of the geopolymer core was assumed to remain constant. Finally, it was accepted that energy deposition occurs within the standard aperture of the Charpy impact test and is assumed to be uniformly distributed over the effective span region. Under these assumptions, the elastic energy density that each phase can store per unit volume is defined by the following expression (Equation (1)) [69]:

(1) $$U_{i}=\frac{\sigma_{f,i}^{2}}{2E_{i}}$$

It is expressed as follows. Here, E_i_ is the modulus of elasticity (Pa) and σ_f,i_ is the flexural strength of that phase (Pa). The total theoretical impact energy of the hybrid sample was calculated by volumetric superposition of the elastic energy contributions of the PLA outer shell and the geopolymer core (Equation (2)) [70]:

(2) $$U_{teo}=\sum_{i}u_{i}V_{i}$$

The volumes of PLA and geopolymer phases are defined as area ratios to the total sample volume, and the effective volume of the PLA phase varies depending on the infill ratio (Equations (3) and (4)) [70,71]:

(3) $$V_{PLA,100}=f_{PLA}V_{total,}\;\;V_{geo,}=f_{geo}V_{total}$$

(4) $$V_{PLA}\left(\varphi \right)=\varphi V_{PLA,100}$$

here, φ represents the infill ratio (20%, 60%, and 100%).

When these expressions are combined, the total theoretical pulse energy, depending on the infill ratio of the hybrid sample, can be expressed by the following general Equation (5) [72,73,74]:

(5) $$U_{teo}\left(\varphi \right)=\;u_{PLA}V_{PLA}\left(\varphi \right)+u_{geo}V_{geo}$$

This model predicts that impact energy will show an approximately linear trend with increasing PLA volume, while the geopolymer core will contribute only minimally to the total energy due to its low fracture toughness. The developed theoretical approach was used to interpret the trending effect of infill density on impact energy, rather than being quantitatively validated with experimental results.

The limitations of the model should be explicitly emphasized. The present approach does not include additional energy-dissipation mechanisms that become active under impact loading, such as plastic deformation, interlayer separation, friction-related energy loss at the PLA–geopolymer interface, crack-path deflection, and local microdamage accumulation. For this reason, the model may quantitatively underestimate the experimental increase in absorbed energy, particularly at higher infill densities. In this context, the model should be regarded not as a quantitative predictive tool, but rather as a simplified reference framework used for the trend-based interpretation of the experimental results.

### 2.5. Qualitative Microscopic Examination of Fracture Surfaces

The fracture surfaces, surface morphology, and interface behavior of PLA and PLA–geopolymer hybrid samples fractured after Charpy impact tests were analyzed under optical and digital microscopes. A ZEISS Axio Scope.A1 optical microscope (ZEISS, Germany) and a Dino Lite AM4515T digital microscope were used for the investigations. The samples were examined directly from the fracture surfaces without any prior preparation (embedding or sectioning). This allowed for clear observation of the crack propagation direction, interlayer separation (delamination), geopolymer phase adhesion characteristics, and pull-out zones on the surface after impact loading. Images were recorded at a magnification range of 20× and a resolution of 2048 × 1536 pixels. For each infill percentage (20%, 60%, and 100%), samples exhibiting the highest energy absorption in the Charpy test were selected, and their fracture surfaces were examined. These studies have enabled the evaluation of microstructural indicators of interface interaction and energy absorption mechanisms in hybrid samples.

### 2.6. Scaling from Sample to New Jersey Barrier

Charpy impact tests quantitatively reveal the energy absorption capacity of PLA–geopolymer hybrid structures. Microstructural observations explain how damage develops through fracture surfaces and interface regions. In this context, the role of the geopolymer phase in the present study is not to act as the primary ductile energy-absorbing phase, but rather to serve as the inner core that provides stiffness, volumetric support, and geometric stability within the hybrid system. During impact, the geopolymer core contributes to a more balanced redistribution of load together with the PLA outer shell and also provides a limited but meaningful contribution to energy dissipation through microcrack formation, local crushing, and interfacial interactions. In contrast, the more pronounced controlled deformation behavior and the dominant energy-absorption mechanisms are primarily associated with the PLA outer shell and the shell–core interaction. Therefore, the fundamental function of the geopolymer in this hybrid architecture is to act as a load-supporting and stabilising core phase. However, in RSBs, performance is not evaluated solely by energy absorption at the sample level. The real determining factor is how energy is distributed within the system and how it accumulates in the barrier body during barrier-vehicle interaction. Therefore, to see the engineering-scale equivalent of the experimental findings, explicit dynamic finite element analyses were performed on a New Jersey-type concrete RSB under a vehicle impact scenario in the next section. Thus, how the trends observed in Charpy tests are reflected in indicators such as internal energy dissipation, deformation propagation, and stress concentration at the barrier scale is examined comparatively. However, this transfer should not be interpreted as a full-scale validation of a directly implementable road safety barrier design, but rather as an exploratory approach aimed at the preliminary engineering-scale assessment of the trends observed at the material scale. For real-world applications, full-scale crash tests, standard-compliance evaluations, durability analyses, and a more detailed investigation of rate-dependent behavior are still required.

### 2.7. FEM Simulation Framework (ANSYS Explicit Dynamics)

The New Jersey-profile concrete barrier is a widely adopted rigid barrier geometry used in median and roadside safety applications. In this study, this barrier geometry was selected to evaluate, at an engineering scale, the potential contribution of the developed PLA–geopolymer hybrid system to impact energy management in RSBs. Internal energy dissipation, deformation propagation, and stress concentration were monitored as primary performance indicators, enabling a combined assessment of impact energy distribution and potential damage zone localization along the barrier.

**Figure 5:**
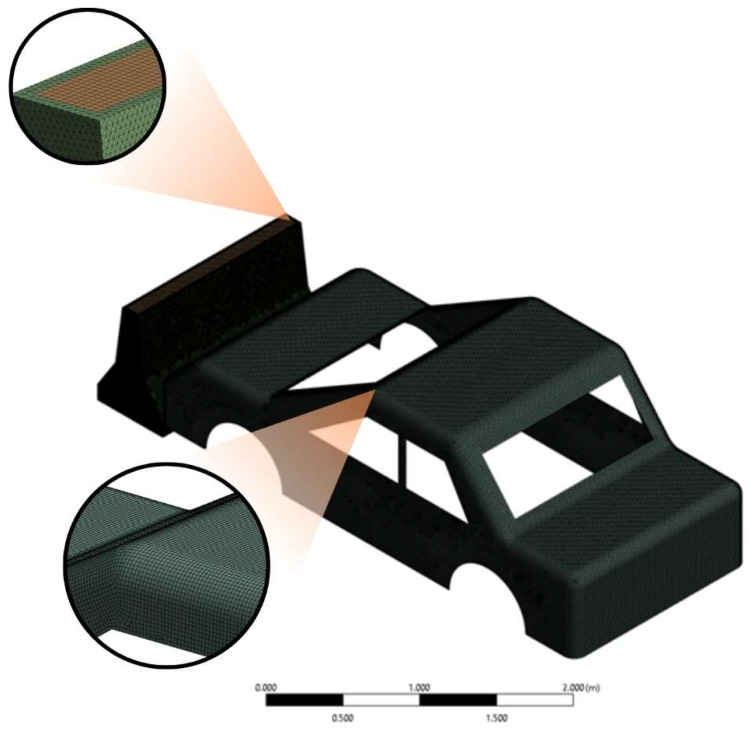
Mesh details.

**Figure 6:**
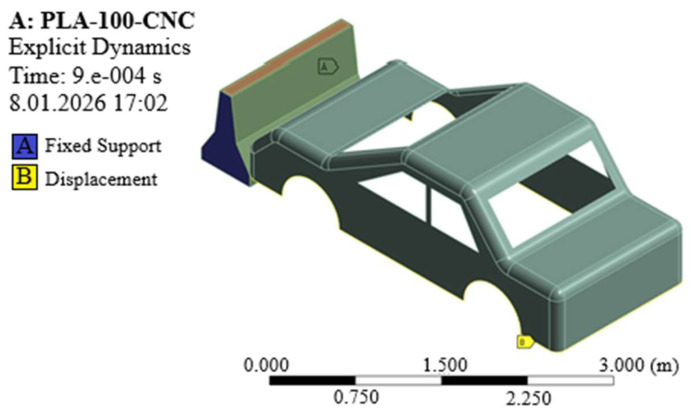
Boundary conditions.

Explicit dynamic finite element analyses were performed in ANSYS Workbench 2021 R2 [75] to simulate the impact behavior of New Jersey-type RSBs comprising geopolymer concrete (CNC) and varying PLA proportions (PLA20–CNC, PLA60–CNC, and PLA100–CNC) under vehicle impact conditions. All components were discretized using 3D solid elements within the explicit dynamics solver, yielding a total model comprising 354,530 elements and 218,610 nodes. A mesh convergence study was conducted by comparing element sizes of 25 mm, 15 mm, and 10 mm; peak internal energy values of 11.00 J, 7.53 J, and 6.80 J were obtained, respectively. High-accuracy finite element representations have been shown to be effective in evaluating local stress concentrations, energy dissipation, and dynamic load transfer in hybrid road safety structures; therefore, in the present study, engineering-scale interpretations are supported within the framework of explicit dynamic FEM. The 15 mm configuration was selected as it demonstrated significantly improved accuracy over the coarser mesh while maintaining acceptable computational efficiency; relative to the finer mesh was considered admissible given the comparative nature of the analysis. Material properties assigned to all barrier configurations were derived directly from the experimental characterization results obtained in this study. It should be noted that rate-independent material models were employed in the present study; the incorporation of rate-dependent constitutive models such as the Johnson–Cook formulation is recommended for future work to further refine the dynamic response predictions. The interface between the PLA infill and the geopolymer core was defined using a perfect bonding (tied contact) formulation, consistent with the mechanical interlocking achieved during the casting process. The contact between the vehicle and the barrier was defined as a frictionless interaction. The frictionless contact assumption was adopted on the basis that inertial forces dominate at the applied impact velocity of 100 km/h, rendering interfacial friction relatively negligible; this condition also represents a conservative lower-bound for energy transfer. The barrier was fully constrained on both lateral faces and the bottom surface, representing a rigid ground connection. For the vehicle, translational degrees of freedom were defined as Ux and Uz free with Uy = 0, and an initial velocity of 100 km/h was applied as a predefined condition at t = 0. The boundary conditions (Table 5) and mesh configuration are illustrated in Figure 5 and Figure 6. The numerical results were evaluated in conjunction with experimental Charpy impact test data to interpret the comparative energy absorption potential of the hybrid structures across different PLA filling ratios.

### 2.8. Statistical Methodology

In this study, experimental data were evaluated within the framework of classical one-way analysis of variance (one-way ANOVA). The basic approach of ANOVA was defined by Fisher and reported as the standard method for comparing group means in single-factor experiments involving three or more levels [76,77]. In this context, the geopolymer matrix, sample geometry, and Charpy V-notch type were kept constant; only the effect of PLA infill percentage (20%, 60%, 100%) on notched impact energy was investigated. Statistical analyses were performed using IBM SPSS Statistics. Descriptive statistics (mean, standard deviation, 95% confidence interval) of notched impact energy were calculated for each infill level; normality was assessed using the Shapiro–Wilk test and Q-Q plots, and homogeneity of variance was assessed using the Levene test. Classical one-way ANOVA relies on the assumptions of normality and homogeneity of variance; violation of these assumptions may affect the validity of the F-statistic [77]. Since the Levene test showed that homogeneity of variance was not achieved, Welch one-way ANOVA was applied for heterogeneous variance conditions, and the Welch F-statistic was used in the interpretations [78,79,80].

When a significant difference was found between groups in ANOVA (*p* < 0.05), post hoc analyses were applied for pairwise comparisons. Since Tukey HSD and similar procedures assume homogeneous variance, they can increase the type I error rate under heterogeneous variance conditions. Therefore, when homogeneity of variance is not ensured, the Games–Howell test, which is compatible with Welch ANOVA and does not require the assumption of equal variances, is preferred; this approach has been shown to provide more robust error control in heterogeneous variances and/or unequal sample sizes [81,82,83,84,85].

In this study, the significance level was set at α = 0.05 for all hypothesis tests. The effect of PLA infill ratio on energy absorption in geopolymer-based, notched Charpy samples was evaluated from both statistical and engineering perspectives using F and *p* values obtained from Welch ANOVA, corrected *p* values obtained from the Games–Howell test, and 95% confidence intervals. Thus, while keeping the material system and sample geometry constant, differences arising solely from infill ratio were systematically revealed within the framework of ANOVA and modern heterogeneity-corrected multiple comparison methods.

## 3. Results and Discussion

### 3.1. PLA and Geopolymer Concrete Material Test Results

The compressive strength results obtained from three different samples tested within the scope of the experimental study are presented in Table 6. According to the results, the compressive strength of the samples ranged from 47.6 MPa to 61.2 MPa, and the average compressive strength was determined to be approximately 54 MPa. These values are consistent with the strength ranges reported in the literature for fly ash and slag-based geopolymer concretes and indicate that the geopolymer core used in the study provides a mechanically adequate carrier phase [86]. This demonstrates that, within a hybrid structure, the geopolymer core can maintain its structural integrity under compressive loads.

The mechanical test results of PLA material show significant differences depending on the infill ratio (Table 7). The results indicate a significant increase in the modulus of elasticity, tensile strength, and flexural strength as the infill ratio of PLA increases. Similar trends have been reported in previous studies on PLA samples produced by the FDM method; it has been emphasized that infill ratio is one of the fundamental parameters determining mechanical performance [52,87]. While low elastic modulus and strength values were observed at 20% infill, a significant improvement in mechanical properties occurred at 60% infill. At 100% infill, PLA samples reached their highest stiffness and strength values. In particular, the tensile strength of 57.3 MPa and the flexural modulus of 3.38 GPa obtained at 100% infill demonstrate that the compact internal structure performs load transfer more homogeneously [4]. Conversely, at low infill ratios, the increase in intramatrix voids leads to stress concentrations and premature failure, resulting in significant decreases in both strength and stiffness. Rockwell hardness results also exhibit a similar trend, revealing that the infill ratio directly affects the surface and volumetric mechanical behavior of PLA.

These mechanical characterization results demonstrate that PLA infill density plays a decisive role not only in static strength but also in energy absorption and damage propagation behavior under impact loads. In this context, the obtained data constitute a fundamental reference for the interpretation of the Charpy impact test results presented in the next section.

### 3.2. Charpy Impact Behavior and Mass Efficiency of PLA–Geopolymer Hybrid Composites

The impact behavior of PLA–geopolymer hybrid composites showed significant differences depending on infill density and notch condition. The average absorbed energy (U) and impact strength (Re) values presented in Table 8 reveal that impact energy generally increases with increasing infill density.

The lowest energy absorption values were obtained in samples with a 20% infill ratio. The average energy was measured as 0.37 ± 0.03 J (Re = 3.74 kJ/m^2^) in notched samples and 0.92 ± 0.12 J (Re = 9.17 kJ/m^2^) in unnotched samples. In this group, energy absorption occurred only at the level of 0.74–1.84%. The high internal void volume at low infill ratios led to localized concentration of stress accumulation during impact and facilitated crack propagation. Caminero et al. (2019) similarly reported that impact strength decreased significantly in FDM-PLA samples with low infill ratios due to weakening of interlayer bonding [52]. A significant improvement was observed at a 60% infill ratio. The average energy was found to be 3.99 ± 0.30 J (Re = 39.93 kJ/m^2^) in notched samples and 7.13 ± 0.30 J (Re = 71.31 kJ/m^2^) in unnotched samples. In this case, the energy absorption rate increased to 7.99–14.28%. This increase can be explained by the geopolymer phase providing more efficient load transfer within the PLA shell. At a medium infill ratio, a balanced structure is formed between both internal stiffness and deformation capacity, leading to more stable crack propagation and increased specific energy absorption capacity. Mechanically, this behavior suggests that the 60% infill structure provides a more favorable balance between stiffness-controlled load transfer and deformation-controlled energy dissipation. In this range, the PLA shell is sufficiently continuous to delay premature crack initiation, while the remaining internal porosity still allows local deformation and crack-path deflection, thereby preventing an excessively brittle response. Similarly, Desole et al. (2024) stated that medium infill ratios exhibit the most efficient behavior in terms of energy absorption per unit mass in 3D-printed PLA metamaterials [53]. Samples with 100% infill showed the highest impact strength. Values of 6.28 ± 0.40 J (Re = 62.79 kJ/m^2^) were obtained in notched samples, and 7.66 ± 0.45 J (Re = 76.61 kJ/m^2^) in unnotched samples. Energy absorption rates reached 12.57–16.00% in this group. The continuity of the geopolymer phase ensured the homogeneous distribution of load transfer between the shell and core, resulting in high rigidity but limited plastic deformation. This indicates the brittle fracture character of the high-density structure. Similarly, Kazantseva et al. (2025) reported that increased rigidity in PLA structures with full infill increased impact strength but reduced ductile deformation [54].

The notch effect was clearly observed in all groups. Stress concentrations at the notch root weakened the crack initiation zone, causing a significant portion of the energy to be expended on crack initiation. This resulted in a decrease in total energy absorption capacity by an average of 25–35% compared to samples without a notch. This behavior is consistent with the classical Charpy fracture mechanism defined in ISO 179-1 [88,89,90]. When evaluated in terms of mass efficiency, samples with a 60% fill rate showed the most balanced performance. In this group, material lightness was maintained while energy absorption capacity remained at a high level [91,92,93]. This has also been numerically confirmed by mass efficiency analyses (Table 9). Mass efficiency is a key performance indicator that expresses the amount of energy absorbed per unit fill rate and quantitatively reveals the balance between the lightness and impact resistance of a material. In this study, the mass efficiency (ME) value was calculated using the following equation (Equation (6)).

(6) $$ME=\frac{U_{mean}}{Infill\;Density\;(\%)}$$

As can be seen from the table, samples with a 60% fill rate exhibited the highest mass efficiency value (ME = 0.066) when normalized according to fill rate. This shows that with approximately 40% less material usage, the energy absorption capacity remains close to 100% fill levels. Therefore, a 60% fill rate represents the region that provides the optimum balance between lightness, strength, and energy absorption. This finding demonstrates that in hybrid PLA–geopolymer systems, porosity is a critical parameter determining not only mechanical strength but also energy absorption efficiency per unit mass.

These findings regarding mass efficiency also highlight the importance of phase interaction and microstructural bonding in hybrid systems. Similarly, Ramos et al. (2022) reported that coating slag-based geopolymer foams with PLA significantly increased dimensional stability and load transfer efficiency, thereby optimizing mechanical performance by preventing the collapse of the porous structure [94]. Kaiser et al. (2013) also showed that impact strength and thermal stability were increased in PLA hybrids reinforced with nanoclay and natural fibers due to the homogeneity of the interface distribution [95].

These results support the finding of a mass efficiency–energy dissipation balance observed in medium-density hybrids. The compatible bonding between the geopolymer core and the PLA shell ensured the continuity of load transfer and controlled crack propagation. Therefore, the medium density range creates an optimum balance point between lightness, deformation capacity, and strength. In conclusion, low-density samples were dominated by interface-controlled brittle fracture and limited load-transfer capacity, whereas high-infill hybrids exhibited higher rigidity, higher absolute energy absorption, and a stronger tendency toward brittle crack propagation. The presence of the geopolymer phase strengthened interfacial bonding in all groups, reducing delamination during crack propagation and increasing energy absorption capacity.

This mass efficiency result offers a direct design implication for RSBs. The aim of barrier systems is not only to steer the vehicle, but also to reduce peak forces and local damage by absorbing impact energy in a controlled manner at the contact zone. In this study, it was observed that a 60% fill rate (ME = 0.066) maintained energy absorption capacity close to 100% fill rate with 40% less material; this indicates that it is a suitable starting point for designing an energy-absorbing “surface element/coating” or “modular, replaceable (sacrificial) interlayer” without unnecessarily increasing the mass of the barrier body. While 100% fill rate increases absolute energy values, it may increase the risk of brittle damage due to higher rigidity and more limited deformation tendency; 20% fill rate may not provide sufficient contribution at the barrier scale due to low energy absorption. Therefore, a 60% infill level in barrier applications can be considered a practical design window in terms of lightness–energy dissipation balance and may form the basis for future approaches such as density grading (e.g., 60% in the contact zone, higher density in the carrier zone).

### 3.3. Theoretical Impact Energy Results and Comparison with Experimental Data

In this section, impact energy values calculated using the theoretical model given in Section 2.5 are analyzed and compared with experimental Charpy results. The energy absorption characteristics of hybrid composites differ significantly due to the change in PLA volume at different infill ratios. This analysis is critical for revealing the elastic and inelastic energy dissipation mechanisms of the hybrid material.

#### Calculation of Theoretical Impact Energy Values

In this study, calculations can be performed according to Equation (5).

$$U_{PLA}=1.27\times 10^{6}\;J/m^{3}$$

$$U_{geo}=2.33\times 10^{3}\;J/m^{3}$$

$$V_{PLA,100}=1.76\times 10^{-6}\;m^{3}$$

$$V_{geo}=1.44\times 10^{-6}\;m^{3}$$

PLA volume is calculated according to the infill ratio given in Equation (4). The data obtained from these calculations are given in Table 10.

According to experimental results, 20% infill hybrid composites exhibit relatively brittle behavior due to the low PLA volume, absorbing an average of 0.36 J of energy. The closeness of the experimental (~0.37 J) and theoretical (~0.45 J) values indicates that fracture occurs via an elastic-brittle mechanism. Since the PLA volume is low, no ductile deformation or layer stretching was observed, and energy storage was largely limited to elastic behavior. Therefore, the theoretical model successfully predicts the actual behavior at low infill levels. The tendency of PLA-based FDM samples to exhibit brittle fracture under impact at low infill ratios has also been reported in the literature. Dave et al. (2023) reported that impact resistance decreased significantly in 3D-printed PLA samples when the infill percentage was low, and that there were also limited traces of plastic deformation on the fracture surface [96]. Similarly, Agrawal et al. (2023) showed that impact resistance and energy absorption of PLA structures increased significantly with increasing infill density, while brittle fracture was dominant at low infill values [97].

Experimental results show an average energy absorption of 3.99 J in 60% infill samples. A significant increase in energy absorption capacity is observed with increasing PLA volume. Although the theoretical and experimental values follow the same increasing trend, the experimental energy is substantially higher than the theoretical prediction, indicating that additional dissipation mechanisms beyond elastic storage become active at 60% infill. These mechanisms likely include local plastic deformation, interface friction, and crack-path deflection, which are not fully captured by the elastic-based model. With a moderate PLA volume, elastic energy is still dominant, but limited ductile deformation, interface microslips, and local layer openings are observed. While these mechanisms slightly alter the theoretical model, the elastic approach still captures the general trend of the 60% infill behavior. This level represents a transition zone between elastic and semi-ductile for the hybrid structure. Recent studies have consistently shown that in PLA structures produced by FDM, increasing the infill ratio increases impact toughness and causes fracture to evolve from a purely brittle character to more complex (partially ductile) mechanisms. Ali et al. (2024) demonstrated that in PLA samples produced with different infill patterns and raster angles, impact toughness increased significantly at medium-high infill ratios and more widespread plastic deformation regions were formed during fracture [98]. Similarly, Tunçel et al. (2024) reported that Charpy impact strength increased with increasing infill density in tough-PLA samples and that the optimum parameter set was strongly related to the infill percentage [99].

These studies support impact energy values observed in 60% infill hybrid samples that are close to, but slightly exceed, the theoretical model. The limited plasticization and interface-related energy dissipation mechanisms that come into play as PLA volume increases mean that the theoretical elastic model remains valid, albeit with a slight deviation. Experimental results show that fully infilled (100% infill) hybrid PLA–geopolymer samples absorb a remarkably high amount of energy, 6.28 J. This is a dramatic increase compared to all other groups.

The experimental value is approximately 3.4 times the theoretical value. The main reasons for this difference are:

- PLA’s ability to induce high-volume plastic deformation,
- Layer-tearing/fiber pull-out occurs between layers as the crack propagates,
- Micro-friction and energy consumption at the PLA–geopolymer interface,
- Elongation of the fracture surface due to deflection of the fracture path,
- Increased microstructural energy dispersion due to 3D printing orientation.

Since the theoretical model only accounts for the elastic component, the experimental energy was much higher when ductility became dominant. 100% infill is the region where ductile fracture is most intense in the hybrid system. Recent microstructure studies have shown in detail that numerous ductile and semi-ductile mechanisms, such as filament/layer debonding, fiber/filament pull-out, matrix cracking, and crack path deflection, are involved in the impact and fracture behavior of fully infilled PLA-based FDM parts. Zhang et al. (2023) reported that the main damage modes in 3D-printed thermoplastic composites are fiber debonding, fiber pull-out, stress bleaching, and matrix cracking; they emphasized that these mechanisms significantly increase energy, especially at high infill ratios [100]. Kargar et al. (2025) showed that filament stretching, interfacial fracture, and fiber stretching are the main mechanisms that directly contribute to fracture toughness in PLA composites reinforced with wood fibers [101].

A more comprehensive review focusing on the pressure orientation, layer interfaces, and ductility-generating mechanisms used in FDM processes Karimi et al. (2024) also emphasizes that fiber pull-out, delamination, and interface fracture are the primary energy-dissipation mechanisms under impact loads [102]. These literature findings explain why the experimental impact energy in 100% infill hybrid PLA–geopolymer samples is much higher than that of the elastic-based theoretical model: while the elastic model only accounts for the elastic stress–strain component, in the actual experimental case, plastic deformation, interface friction, and microstructural damage mechanisms create an additional energy absorption capacity.

When the theoretical and experimental results are evaluated together, the model successfully captures the overall trend that impact energy increases with increasing infill density, but it systematically underestimates the experimental values. This deviation arises from non-elastic energy-dissipation mechanisms such as plastic deformation, interlayer opening, micro-friction at the interface, and crack-path deflection, which become increasingly active, particularly at medium and high infill densities. Therefore, within the scope of the present study, the role of the theoretical model is not to provide a one-to-one quantitative prediction that exactly matches the experimental data, but rather to offer a baseline comparative framework that reveals the underlying elastic contribution and highlights the importance of the additional energy-absorption mechanisms observed experimentally.

### 3.4. Qualitative Microscopic Observations of Fracture Surfaces

**Figure 7:**
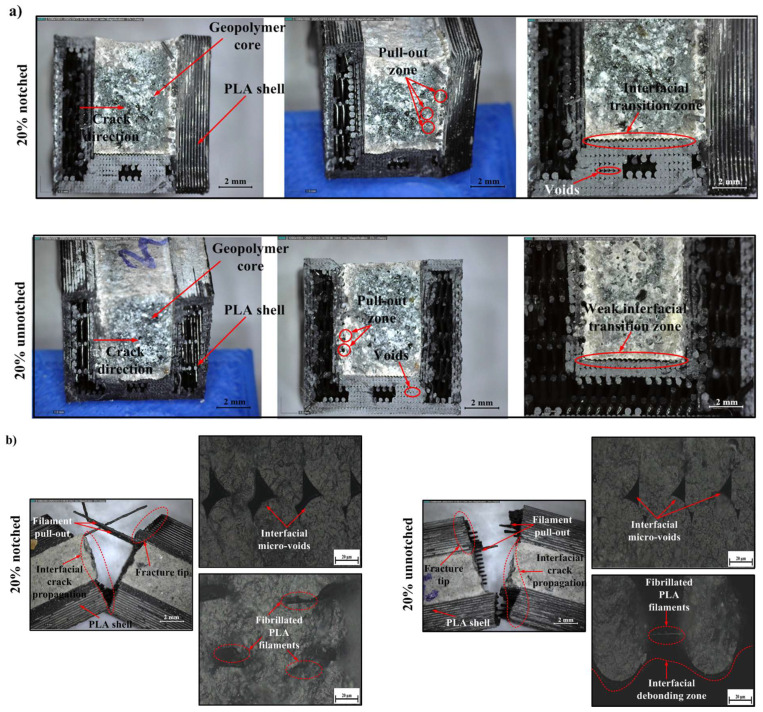
Digital and optical micrographs of 20% infill specimens, (**a**) fracture surfaces of notched (top) and unnotched (bottom) samples, and (**b**) interfacial regions showing filament pull-out, micro-voids, and debonding within the PLA–geopolymer interface.

In samples with a low infill percentage (20%), fracture surfaces generally exhibited brittle behavior (Figure 7). The following observations are presented as qualitative fractographic evidence of damage evolution at the fracture surface and interface, with particular attention to features such as interfacial voids, filament pull-out, debonding, crack deflection, and local plastic deformation. In notched samples, the crack initiation point was oriented from the inner surface of the PLA shell towards the geopolymer core due to stress concentrations at the root of the notch, and typical interfacial crack propagation was observed. The porous interfacial transition zone (ITZ) and interfacial voids in these regions indicate weak bond strength along the interface and easy crack propagation along this line. Insufficient wetting and lack of adhesion created a predisposition to filament pull-out and local delamination zones. This is consistent with the measured low absorbed energy (U) and impact strength (Re) values. Digital microscope images clearly show filament pull-out zones and interfacial micro-voids. These micro-voids demonstrate both a lack of cohesion at the material interface and insufficient interlayer adhesion in FDM-based fabrication. Perez et al. (2021) similarly reported that micro-voids at the interface in PLA samples produced with FDM reduce fracture resistance and that a significant portion of the energy is expended in the crack initiation process [103].

While the fracture surface in unnotched samples exhibited similar morphological characteristics, the crack line followed a more irregular and longer path due to the elimination of the notch effect. In this case, microplastic deformation, fibrillated PLA filaments, and pull-out mechanisms provided additional energy dissipation, albeit at a low level. The fibrillated filament structures observed in optical microscope images indicate that the polymer underwent local plastic deformation, but the low total energy absorption reveals that this deformation was only local. These findings are consistent with the literature, indicating that low-infill-rate FDM structures exhibit a high tendency for delamination, weak mechanical bonding, and limited impact resistance [97]. Similarly, Korniejenko et al. (2022) stated that weak interfacial transition regions in polymer–geopolymer composites reduce energy absorption capacity and affect fracture behavior in a brittle direction [104]. In conclusion, the fracture behavior in samples with a 20% fill rate can be largely characterized by brittle fracture along the interface. The load transfer capacity of the PLA shell remained limited, and fracture energy was absorbed at a low level due to the weak bonding structure and voids at the interface.

**Figure 8:**
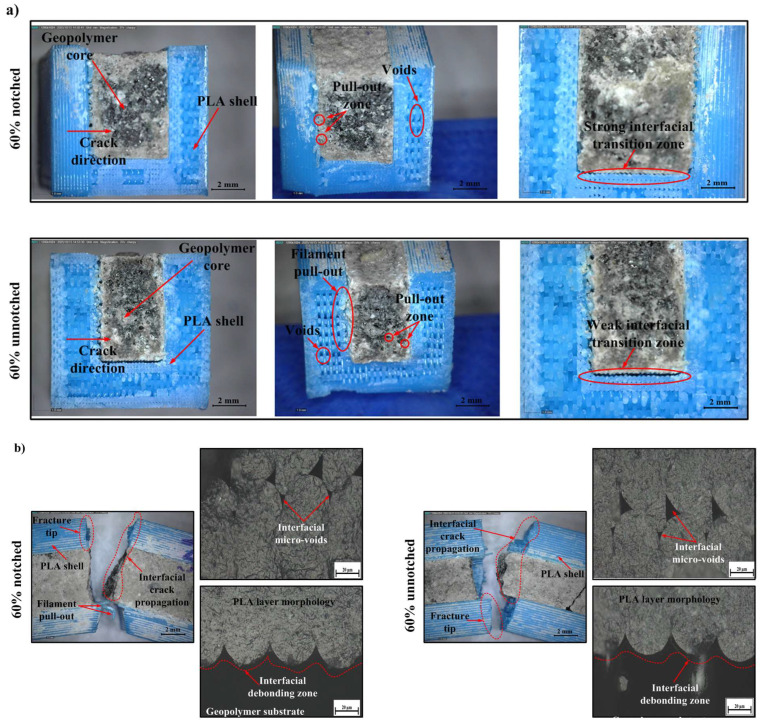
Digital and optical micrographs of 60% infill specimens, (**a**) fracture surfaces of notched (top) and unnotched (bottom) samples, and (**b**) interfacial regions showing filament pull-out, micro-voids, and debonding within the PLA–geopolymer interface.

In samples with 60% infill, the fracture surface exhibited a more curved and rough topography compared to 20%. This indicates that crack propagation shows a tendency towards deflection along the interface and that fracture energy is distributed more homogeneously (Figure 8). The presence of limited filament pull-out areas along with a compact ITZ (strong bonding) structure was observed in the interface regions. This morphology shows that load transfer is enabled and the PLA shell interacts better with the geopolymer core. Similarly, Tunçel (2024) reported that medium infill ratios increase impact strength and improve stress distribution in PLA structures produced with FDM [99].

Local filament retractions and low-density interfacial voids were observed in the unnotched samples, but the ITZ is largely a tight and continuous structure. This can be explained by the fact that energy propagates through the interface, not by completely breaking off, but by changing direction in places during fracture. Thus, microplastic deformation and mechanical interlocking play a role simultaneously (Figure 8). Optical microscope images show that stronger mechanical interlocking occurs between the PLA sheets and the geopolymer substrate, and that microvoids are less dense. This type of quasi-ductile fracture behavior is consistent with cases in the literature where moderate infill ratios correspond to optimum energy absorption capacity [105]. In conclusion, this micromorphology obtained at 60% infill indicates that mechanical bonding, energy dissipation, and crack deflection mechanisms occur in a balanced manner, thus demonstrating that this percentage is the optimum infill level in terms of mechanical performance.

**Figure 9:**
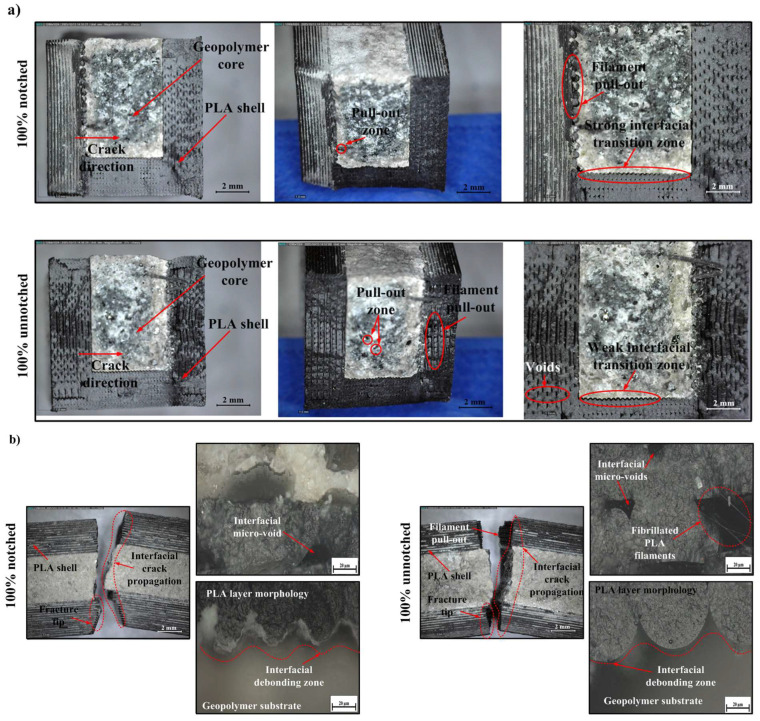
Digital and optical micrographs of 100% infill specimens, (**a**) fracture surfaces of notched (top) and unnotched (bottom) samples, and (**b**) interfacial regions showing filament pull-out, micro-voids, and debonding within the PLA–geopolymer interface.

In samples with full infill (100%), a dense and compact ITZ structure with a limited number of microvoids was observed along the interface. This structure indicates a prominent combination of high rigidity and low ductile deformation. The fracture surface exhibited a more planar and brittle character; this indicates that energy was largely dissipated during the crack initiation and rapid crack propagation process (Figure 9). The literature also reports that at high infill ratios, interlayer fracture toughness increases while ductile energy absorption capacity decreases [106]. In notchless specimens, the presence of a compact ITZ, limited filament pull-out, and rare interfacial voids is noteworthy. Crack propagation occurred as planar fracture patterns progressing in short jumps rather than long-range delamination. This morphology supports rigid and brittle behavior, consistent with the high Re values measured. Furthermore, the higher fracture initiation energy and increased total energy absorption in the unnotched structure are consistent with the impact mechanism defined in the ISO 179-1 standard [66,107]. In notched samples, it was observed that both the integrity of the PLA layers and the adhesion capacity of the geopolymer substrate were preserved during interfacial crack propagation. The low number of micro-voids and the locally fibrillated PLA filaments indicate that limited plastic deformation occurs at the interface and that energy is effectively dissipated. This behavior confirms that the fully filled PLA matrix plays a dominant role in load transfer and that fracture occurs through controlled crack propagation (Figure 9). However, localized filament-geopolymer debonding was observed in some areas. This situation can be attributed to the different deformation rates occurring at the interface due to the difference in elastic modulus of the two materials.

As the fill rate increased, the fracture surface morphology and energy absorption mechanism changed significantly. At 20% fill rate, fracture largely progressed along the interface due to the weak and porous ITZ structure; prominent interfacial voids, filament pull-out, and fibrillated PLA filaments revealed a brittle fracture character [108]. At 60% infill, the fracture surfaces acquired a rougher and more complex structure; the compact ITZ, partial pull-out, and crack deflection traces indicated that energy was dissipated more evenly in both interface and interlayer regions [109]. This structure corresponds to the optimum energy absorption level exhibiting quasi-ductile behavior. At 100% fill, the ITZ becomes denser and more continuous, reducing the formation of micro-voids and debonding zones; however, fracture occurs more rigidly and planarly, limiting the ductile deformation capacity [110]. Overall, brittle fracture mode was observed at low fill level, semi-ductile at medium fill level, and rigid/brittle fracture mode at full fill level; this indicates that 60% fill level provides optimum performance in terms of mechanical bonding and energy dissipation.

### 3.5. FEM Simulation Results

#### 3.5.1. Impact Process and Energy Transfer

In this section, the outputs obtained from explicit dynamic analyses conducted on the New Jersey-type concrete barrier are considered together to interpret the collision performance at the barrier scale. The barrier’s energy absorption level during a collision is evaluated based on how much of the kinetic energy entering the system is converted into internal energy within the barrier. It reveals how deformation propagates along the barrier body, the path along which energy is transferred, and where it tends to accumulate. In addition, stress concentrations indicate critical areas in terms of crushing or crack formation, helping to identify potential points of damage initiation.

**Figure 10:**
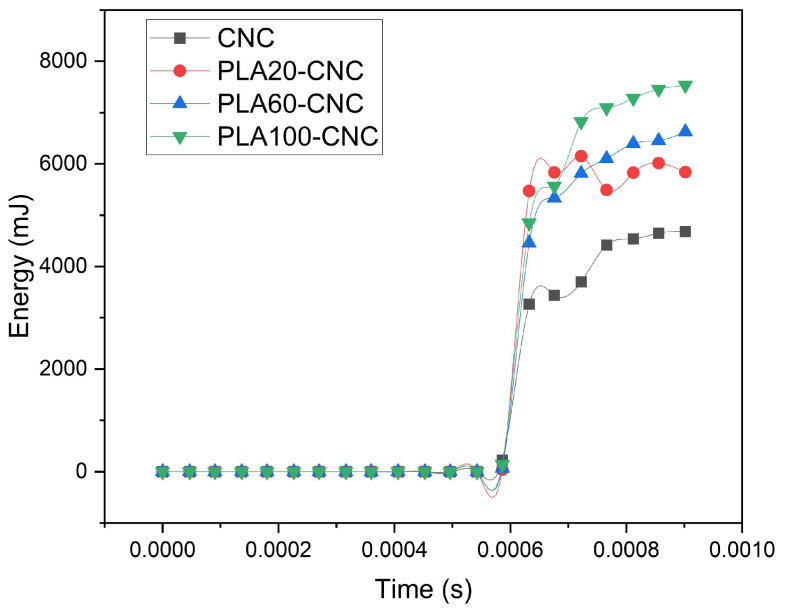
Time-dependent energy absorption.

When examining the collision process, it was observed that the vehicle–barrier interaction commenced at approximately t ≈ 5.86 × 10^−4^ s, and from this point onwards, the vehicle’s kinetic energy rapidly decreased and was converted into internal energy within the barrier body. The fact that the internal energy values were zero in all models up to this point indicates that the system was numerically stable prior to contact. With the onset of contact, the internal energy values exhibited a rapid and regular increase in all barrier types, and no numerical oscillations or instability were observed in the energy–time curves. The energy absorption capacities of the barriers were compared based on the total internal energy values reached at the end of the collision process. The internal energy values obtained at t = 9.02 × 10^−4^ s were 4678 mJ (4.68 J) for CNC, 5838.5 mJ (5.84 J), 6626 mJ (6.63 J) for PLA60–CNC, and 7531.5 mJ (7.53 J) for PLA100–CNC. The results obtained reveal that the barrier’s capacity to absorb collision energy increases significantly with increasing PLA content. Specifically, it was determined that the PLA100–CNC model absorbed approximately 61% more energy compared to the traditional CNC barrier. This indicates that the PLA additive positively affects the collision performance by increasing the barrier’s capacity for energy dissipation and damage propagation under impact (Figure 10).

The temporarily higher energy absorption of PLA20–CNC in the 0.0006–0.0007 s interval is attributed to its stiffer geopolymer matrix, which promotes rapid stress wave propagation upon initial impact; beyond this phase, the greater viscoelastic deformation capacity of PLA60–CNC and PLA100–CNC enables sustained energy dissipation, resulting in higher total absorbed energy.

#### 3.5.2. Deformation Behavior

When examining the deformation contours, it was observed that shape changes were limited in the CNC model and the barrier exhibited more rigid behavior. In contrast, in models containing PLA, more pronounced deformation occurred in the impact zone and spread over a wider area along the barrier. The presence of the PLA shell contributed to the deformation developing in a more controlled manner, thereby supporting more effective dissipation of the collision energy within the barrier. From a mechanical standpoint, this indicates that the PLA-containing configurations redistribute impact-induced strain over a wider region instead of concentrating it at a limited contact zone. This interpretation is consistent with the specimen-level observations, where improved interface continuity and crack deflection at intermediate-to-high infill levels promoted more stable damage evolution and reduced the tendency for abrupt localized failure.

#### 3.5.3. Stress–Strain Distributions

**Figure 11:**
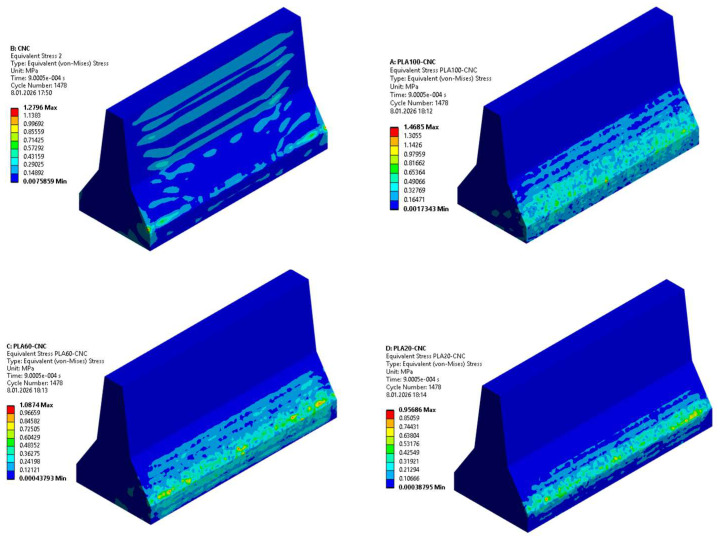
Stress Distributions in Barriers.

**Figure 12:**
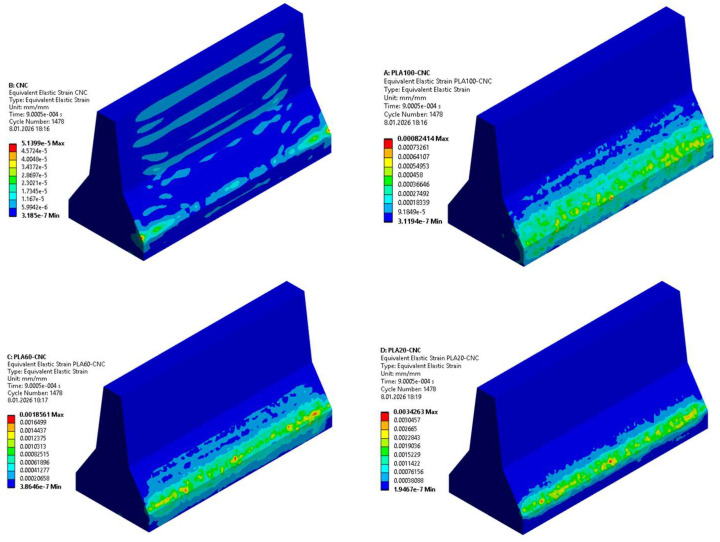
Shape Change Distributions at the Barrier.

When comparing the equivalent elastic strain contours of PLA-CNC and reference CNC barriers, significant differences in deformation mechanisms are observed. In the CNC model, deformations are predominantly localised at the base corners and impact contact zone, while a large portion of the barrier body remains at low strain levels. The concentration of maximum strain values in limited areas indicates that the geopolymer concrete exhibits a more brittle character and that the impact energy accumulates in localised regions. In contrast, in the PLA100–CNC model, although maximum strain values again occur in the impact region, it is observed that the strain area is spread over a wider region along the barrier. This situation reveals that deformation is not concentrated at single points, but rather distributed more homogeneously through the PLA layer. Furthermore, the low strain levels in the geopolymer concrete body in the PLA100–CNC model indicate that deformation is largely concentrated in the PLA component and that impact energy is damped by ductile plastic deformation mechanisms. These comparative results clearly demonstrate that the PLA additive reduces the risk of brittle damage and increases the energy absorption capacity by redistributing stress and deformation within the barrier system (Figure 11 and Figure 12).

The peak equivalent (Von Mises) stress values extracted from the FEM results were 1.28 MPa, 0.96 MPa, 1.09 MPa, and 1.47 MPa for CNC, PLA20–CNC, PLA60–CNC, and PLA100–CNC configurations, respectively. Notably, the highest stress magnitude observed in PLA100–CNC is accompanied by the widest distribution area, indicating that greater stress is sustained across a larger volume—a behavior consistent with enhanced energy dissipation rather than localised failure. These quantitative results further confirm the transition from brittle, localised damage in the CNC barrier to a more distributed and ductile response in the hybrid configurations.

### 3.6. Statistical Outcomes

The notched Charpy impact energy results for PLA infill ratio reveal a consistent upward trend in absorbed energy as infill increases. The average impact energy was approximately 0.37 J at 20% infill, approximately 3.99 J at 60% infill, and approximately 6.28 J at 100% infill; thus, increasing the amount of PLA in the geopolymer matrix significantly increased the energy absorption capacity of the samples. The Levene test showed that the assumption of equal variance between groups was not met (*p* = 0.001). Therefore, in addition to the classical one-way ANOVA, a Welch one-way ANOVA sensitive to variance heterogeneity was applied to evaluate the difference between groups. The analysis showed that the infill ratio had a statistically significant effect on the impact energy [classical ANOVA: F(2, 13) = 9.340, *p* = 0.003; Welch ANOVA: F(2, 5.334) = 16.540, *p* = 0.005].

To determine which groups showed the most significant differences in infill levels, the Games–Howell post hoc test, which does not require the assumption of equal variances, was used. The results showed a statistically significant difference between samples with 20% and 100% infill ratios (*p* = 0.011); in other words, fully occupied samples absorbed approximately 5.9 J more energy on average compared to low-infill samples. In contrast, the *p*-values for the 20–60% (*p* = 0.153) and 60–100% (*p* = 0.470) pairs remained above the 0.05 threshold, and the differences between these groups were not found to be statistically significant. Overall, when the Welch ANOVA and Games–Howell results are considered together, it can be said that increasing the PLA infill ratio strongly increases impact energy absorption when the geopolymer matrix is kept constant, but the most significant and statistically consistent divergence occurs between low infill (20%) and high infill (100%).

From an engineering perspective, the lack of a significant difference between 60% and 100% infill ratios (*p* = 0.470) is particularly important. In the mass efficiency analysis, it was observed that samples with a 60% infill ratio exhibited the highest performance in terms of energy absorbed per unit infill ratio; ME values were calculated as 0.046, 0.066, and 0.063 for 20%, 60%, and 100%, respectively. Thus, with approximately 40% less PLA usage, a 60% infill ratio achieves statistically similar impact energy levels to 100% infill, while offering a higher energy absorption capacity per unit mass. This result supports both the mass efficiency findings obtained from Charpy tests and the targeted lightness–energy absorption balance at the barrier scale, highlighting a 60% infill ratio as a rational and efficient engineering target for energy-absorbing hybrid layer design in RSBs.

## 4. Conclusions

This study investigates the energy absorption behavior of hybrid composites with a polylactic acid (PLA) outer shell produced using 3D printing and a geopolymer-filled inner core under impact loading through a three-component evaluation framework. Within this scope, the system was evaluated through theoretical energy absorption calculations, Charpy impact tests, and finite element analysis (FEA), and the feasibility of an alternative energy-absorbing material architecture suitable for use in lightweight, modular, and environmentally sustainable RSBs was investigated.

- Experimental findings have shown that the void ratio is decisive for the fracture mode, ITZ (interfacial transition zone) character and specific energy absorption (SEA).
- At a 20% filling ratio, fracture predominantly propagated along the interface due to the porous and weak ITZ, with pronounced filament pull-out and interfacial voids observed, resulting in brittle fracture behavior with limited energy dissipation.
- At 60% filling, the ITZ attained a more compact structure, and a quasi-ductile fracture occurred due to the simultaneous effect of crack deflection and mechanical interlocking mechanisms. This configuration provided optimal performance in terms of SEA.
- At 100% filling, the ITZ gained continuity, and despite the reduction in microvoids, fracture occurred more rigidly and planar, with limited ductile deformation capacity.
- Microstructural investigations revealed that the PLA outer shell played an active role in load transfer, while the geopolymer core dissipated most of the energy during impact through microcrack formation, void collapse, and internal friction. Thus, the shell–core interaction provided controlled energy distribution, preventing sudden and brittle failure.
- Finite element analyses have revealed that New Jersey-type RSBs based on PLA-modified geopolymer concrete produce higher internal energy conversion during impact, thereby dissipating energy more effectively within the barrier.
- When compared to the reference geopolymer concrete barrier (CNC), the stress and strain distributions indicate that impact loads are directed towards the PLA layer, resulting in more controlled deformation and damping in the hybrid system. These results support the notion that the hybrid barrier can exhibit more effective collision performance under the specified boundary conditions and loading scenarios.
- Overall, the findings indicate that PLA–geopolymer hybrid systems offer a viable material platform for lightweight, energy-absorbing RSBs that meet both structural strength and environmental sustainability criteria.
- The fact that a 60% filling ratio produces optimal SEA performance points to design strategies that maximise energy dissipation capacity while reducing material usage in 3D printing-based production.
- The industrial waste-derived binder structure of geopolymer reduces the carbon footprint, while the biodegradable character of PLA increases the sustainability potential of the system. Thus, hybrid systems are positioned as a lighter, modular, repairable alternative with a lower environmental impact level compared to traditional metal barriers.

Future studies will systematically investigate the effects of parameters such as the mineralogical composition of geopolymer components, additive types, layer orientations, and PLA shell thickness. This, combined with FEM-based optimisation studies, will enable more accurate modelling of energy dissipation behavior. In this context, new-generation engineering approaches are being pioneered for the development of high-performance, sustainable RSBs that can be produced on-site using 3D printing technologies. Furthermore, the scalability of the additive manufacturing process to full-scale road barrier production and the extension of the numerical model to more complex impact scenarios will also be addressed in future work. Additionally, a comparative assessment of the proposed hybrid barrier system against alternative energy-absorbing configurations, such as foam-filled and honeycomb-core barriers, is planned for future studies.

## Figures and Tables

**Table 1:** Mechanical and printed properties of PLA filament [59].

Properties	PLA
Specific gravity (g/cm^3^)	1.2
Tensile strength (MPa)	72
Elongation to fracture (%)	11.8
Print temperature (°C)	210–260
Print speed (mm/s)	40–100

**Table 2:** Geopolymer mixture.

Component	Quantity (g)	Weight (%)
Fly Ash (YFC)	2250	33.82
Water	105	1.58
Plasticizer	57	0.86
Aggregate	3100	46.60
Silicate	804	12.09
Hydroxide	322	4.84

**Table 3:** Bambu Lab X1 Carbon technical specifications [64].

Parameters	Value
Print Resolution	±0.01 mm
Layer Resolution	0.05–0.30 mm
Print Speed	10–500 mm/s
Max Temperature Extruder	300 °C
Min Temperature Extruder	110 °C

**Table 4:** Process variables and their levels.

Parameters	Value
Base Speed (mm/s)	60
Travel Speed (mm/s)	80
Layer Height (mm)	0.25
Printing Temperature (°C)	220
Platform Temperature (°C)	60
Nozzle Diameter (mm)	0.4
Slice Profile	Fine
Infill Pattern	Line
Infill Density	20, 60 and 100%

**Table 5:** Boundary conditions and loading parameters of the FEM simulation.

Parameter	Definition/Value
Barrier Constraints	Both lateral faces and bottom surface—fully fixed
Vehicle—Ux	Free
Vehicle—Uy	Constrained (=0)
Vehicle—Uz	Free
Contact Definition	Frictionless (vehicle–barrier interface)
Initial Velocity	100 km/h (applied at t = 0)

**Table 6:** Compressive strength of geopolymer specimens.

Specimen	Compressive Strength (MPa)
1	53.3
2	61.2
3	47.6

**Table 7:** Mechanical properties of PLA.

Infill (n = 3)	Elasticity Modulus E (GPa)	Tensile Strength (MPa)	Yield Strength (MPa)	Bending Modulus (GPa)	Bending Strength (MPa)	Rockwell R Hardness
20%	0.094	5.1	4	0.135	6.9	9.4
60%	0.846	26.6	21	1.217	35.9	48.8
100%	2.35	57.3	45.2	3.38	77.4	105

**Table 8:** Average impact energy (U) and impact strength (Re) of PLA–geopolymer hybrid composites at different infill densities.

Infill Density (%)	Specimen Type	n	Absorbed Energy (%)	U (J) (Mean ± SD)	Re (kJ/m^2^) (Mean)
20	Notched	6	0.74	0.37 ± 0.03	3.74
20	Unnotched	6	1.84	0.92 ± 0.12	9.17
60	Notched	6	7.99	3.99 ± 0.30	39.93
60	Unnotched	6	14.28	7.13 ± 0.30	71.31
100	Notched	6	12.57	6.28 ± 0.40	62.79
100	Unnotched	6	16.00	7.66 ± 0.45	76.61

**Table 9:** Mass efficiency (ME) values of PLA–geopolymer hybrid specimens at different infill ratios.

Infill Density (%)	U (J, Notched)	Mass Efficiency (ME)
20	0.92	0.046
60	3.99	0.066
100	6.28	0.063

**Table 10:** Theoretical–experimental energy comparison.

Infill (%)	Theoretical Energy (J)	Experimental Energy (J)	Difference	Comment
20	0.45	0.37	Very small	Brittle fracture +model compatible
60	1.35	3.99	Small	Limited ductile behavior
100	2.25	6.28	Very large	Plastic deformation + interface friction

## Data Availability

The original contributions presented in the study are included in the article. Further inquiries can be directed to the corresponding author.
